# Health-care users, key community informants and primary health care workers’ views on health, health promotion, health assets and deficits: qualitative study in seven Spanish regions

**DOI:** 10.1186/s12939-017-0590-2

**Published:** 2017-06-13

**Authors:** Mariona Pons-Vigués, Anna Berenguera, Núria Coma-Auli, Haizea Pombo-Ramos, Sebastià March, Angela Asensio-Martínez, Patricia Moreno-Peral, Sara Mora-Simón, Maria Martínez-Andrés, Enriqueta Pujol-Ribera

**Affiliations:** 1 Institut Universitari d’Investigació en Atenció Primària Jordi Gol (IDIAP Jordi Gol), Av. Gran Via de les Corts Catalanes 587, àtic, 08007 Barcelona, Spain; 2 Universitat Autònoma de Barcelona, Bellaterra (Cerdanyola del Vallès), Spain; 3 Universitat de Girona, Girona, Spain; 4 Primary Care Research Unit of Bizkaia, Basque Health Service-Osakidetza, Bilbao, Spain; 5 Primary Care Research Unit of Mallorca, Balearic Health Services-IbSalut, Palma, Spain; 6 Instituto de Investigación Sanitaria de Palma, Palma, Spain; 7 Aragon Institute for Health Research (IIS Aragon), Zaragoza, Spain; 8 Department of Psychology and Sociology, University of Zaragoza, Zaragoza, Spain; 9 Instituto de Investigación Biomédica de Málaga (IBIMA), Distrito Sanitario Málaga-Guadalhorce, Málaga, Spain; 10 Primary Care Research Unit, The Alamedilla Health Centre, Castilla and León Health Service (SACyL), Biomedical Research Institute of Salamanca (IBSAL), Salamanca, Spain; 11 Social and Health Care Research Centre, University of Castilla- La Mancha, Cuenca, Spain

**Keywords:** Health, Health assets, Health promotion, Patient participation, Primary Health Care, Qualitative research

## Abstract

**Background:**

Although some articles have analysed the definitions of health and health promotion from the perspective of health-care users and health care professionals, no published studies include the simultaneous participation of health-care users, primary health care professionals and key community informants. Understanding the perception of health and health promotion amongst these different stakeholders is crucial for the design and implementation of successful, equitable and sustainable measures that improve the health and wellbeing of populations. Furthermore, the identification of different health assets and deficits by the different informants will generate new evidence to promote healthy behaviours, improve community health and wellbeing and reduce preventable inequalities. The objective of this study is to explore the concept of health and health promotion and to compare health assets and deficits as identified by health-care users, key community informants and primary health care workers with the ultimate purpose to collect the necessary data for the design and implementation of a successful health promotion intervention.

**Methods:**

A descriptive-interpretive qualitative research was conducted with 276 participants from 14 primary care centres of 7 Spanish regions. Theoretical sampling was used for selection. We organized 11 discussion groups and 2 triangular groups with health-care users; 30 semi-structured interviews with key community informants; and 14 discussion groups with primary health care workers. A thematic content analysis was carried out.

**Results:**

Health-care users and key community informants agree that health is a complex, broad, multifactorial concept that encompasses several interrelated dimensions (physical, psychological-emotional, social, occupational, intellectual, spiritual and environmental). The three participants’ profiles consider health promotion indispensable despite defining it as complex and vague. In fact, most health-care users admit to having implemented some change to promote their health. The most powerful motivators to change lifestyles are having a disease, fear of becoming ill and taking care of oneself to maintain health. Health-care users believe that the main difficulties are associated with the physical, social, working and family environment, as well as lack of determination and motivation. They also highlight the need for more information. In relation to the assets and deficits of the neighbourhood, each group identifies those closer to their role.

**Conclusions:**

Generally, participants showed a holistic and positive concept of health and a more traditional, individual approach to health promotion. We consider therefore crucial to depart from the model of health services that focuses on the individual and the disease toward a socio-ecological health model that substantially increases the participation of health-care users and emphasizes health promotion, wellbeing and community participation.

## Background

The concept of health has changed in accordance with the knowledge, beliefs and values of each historical and sociocultural period [1]. The traditional approach that equates health with absence of disease and focuses on the individual has progressed toward a more dynamic, multicomponent, positive, holistic and collective definition [2, 3] that considers health a universal human right. The Constitution of the World Health Organization (WHO) (1946) states that “health is a state of complete physical, mental and social well-being, and not merely the absence of disease or infirmity” [4]. According to the general practitioner Jordi Gol, “health is living with autonomy, solidarity and happiness” [5]. This comprehensive view of health implies an essential role for health promotion (HP), where health assets (HA) become centre stage [6]. The Ottawa Charter [4] declares that HP is “the process of enabling people to increase control over their health and its determinants, and thereby improve their health. It moves beyond a focus on individual behaviour towards a wide range of social and environmental interventions” [4].

The conception of HP is closely linked to the notion of community action, since it centres on the population, raising awareness and encouraging community responsibility and involvement [7]. In addition, HP is a field closely related to the principles and development of Primary Health Care (PHC). Indeed, the accessibility, follow-up and continuity of primary care services and their presence in the community constitute the ideal context to offer integrated and person-focused care and to implement HP activities [8]. However, the incorporation of HP interventions in the daily practice of PCH remains a challenge, with barriers such as heavy workload, time constraints and the beliefs of professionals and patients on HP, as shown in two qualitative syntheses [9, 10].

The implementation of HP must take into account the HA of the community [11]. A ‘health asset’ can be defined as any factor or resource that enhances the ability of individuals, groups, communities, populations, social systems and institutions to create, maintain and sustain health and well-being and reduce health inequities. These assets can operate at the individual, group, community and population level as protective factors against stress, improving individual and community health and wellbeing and reducing preventable inequalities [6, 12]. Despite accumulated information on the performance of HA, improved, evidence-based data are currently needed [13], in particular regarding the articulation of asset mapping and public health efforts within a given social context [14].

Health-related behaviours are strongly linked to the social, cultural, economic and structural factors that people experience throughout their lives [15]. Consequently, understanding the baseline concept of health and HP of the different stakeholders is crucial for the design and implementation of successful and sustainable measures to improve the health and wellbeing of populations. The importance of this knowledge is underscored by the current context of financial crisis, population aging and raise of chronic diseases. Indeed, most chronic conditions and their complications are highly preventable with the implementation of HP and disease prevention strategies [4].

This qualitative study corresponds to a section of the results of the second phase (development of the intervention) of the EIRA Project, which follows the UK Medical Research Council framework for complex interventions [16–18]. The objective of the EIRA Project is to design, carry out and evaluate a complex, multi-risk intervention to reduce tobacco consumption, poor adherence to the Mediterranean diet, insufficient physical activity, cardiovascular risk factors and risk of depression in people between 45 to 75 years that contact primary care services with at least two of these behaviours or risk factors, in order to develop health-promoting behaviours to improve quality of life and for the prevention of the most common chronic diseases. Participants receive social prescribing and individual recommendations on their behaviour and risk factors and they also attend group sessions in the PHC setting [19].

For the design of complex interventions that involve HP activities, in-depth knowledge of the context as well as the involvement and cooperation between health care users and health care professionals are crucial [18]. In research, public involvement currently implies conducting research ‘with’ or ‘by’ the public, rather than ‘to’, ‘about’ or ‘for’ the public [18, 20]. However, in our context the public is scarcely involved in the formulation of research that might have a direct impact on their lives. As exemplified by the ongoing EIRA project, we strongly believe that it is essential to take into account the discourses of all stakeholders with regard to health, HP, HA and health deficits to successfully implement the most adequate, acceptable, equitable and sustainable strategies for HP and well-being in each context since attitudes, behaviours and practices differ in accordance with the interpretation of concepts [2]. Although some articles have analysed the definitions of health and HP from the perspective of health-care users [15, 21, 22] and health care professionals [2, 23–25], we have not identified prior studies with the simultaneous participation of health-care users, PHC professionals and key community informants. Furthermore, comparing the different HA and deficits identified by the different informants will generate new evidence to facilitate and promote healthy behaviours and to improve the health and wellbeing of populations.

The aim of this study is to explore the concept of health, HP and related activities and to compare HA and deficits as identified by health-care users, key community informants and PHC workers in 7 Spanish regions. We consider this knowledge indispensable for the successful design and implementation of an equitable complex health promotion intervention in primary care.

## Methods

### Design

Descriptive-interpretive qualitative research [26] to understand the concept and relevance of health and HP in the daily lives of health-care users, key community informants and PHC workers. In addition, HA and deficits were identified to explore and compare the elements singled out by each type of informant in very diverse geographical and socioeconomic contexts. The framework for this research is HP based on the salutogenic paradigm and HA.

### Setting and study population

14 primary care centres (PCC) from 7 Spanish regions (2 PCC per region) participated: Andalusia (Malaga), Aragon (Zaragoza), Balearic Islands (Palma de Mallorca), Basque Country (Vitoria-Gasteiz), Castilla-La Mancha (Cuenca), Castilla-Leon (Salamanca) and Catalonia (Barcelona).

The study population were: i) health-care users from 45 to 75 years from participant PCC (target population of the EIRA Project); ii) key informants with in-depth knowledge of the community context (community workers and health workers with a managerial role or working directly in the community); and iii) workers from participating PCC (professionals based in the PCC, including social workers and administrative staff).

### Sample design and participant selection strategy

Participants were selected by means of theoretical sampling. The profiles of the informants were defined to represent different groups of the study population and their discourse variability, with the objective to achieve richness of information and in-depth understanding of the phenomenon [27, 28]. Table 1 shows the variables used to define the informants’ profiles.

**Table 1:** Variables considered for developing the informants’ profiles

Participants	Sampling attributes
Health-care users (object of the EIRA intervention)	Geographical area
	Gender
	Age
	Educational level
Key community informants (with in-depth knowledge of the context and population object of the intervention)	Geographical area
	Community workers or health workers with a managerial role or working directly in the community
	Professional profile (Representatives of associations, social groups, residents’ association, sports centres, councillors for community public health, community pharmacies, primary care managers)
	Gender
	Age
Primary health care workers	Geographical area
	Professional profile (administrative staff, nurses, physicians and social workers)
	Gender
	Age
	Years of professional experience

In agreement with the Data Protection Law, health-care users received a phone call from their named, accountable health care professionals to explain the objectives of the study and were invited to participate. The voluntary aspect of participation was emphasized. Health-care users that showed an interest in participating and that gave their consent to be contacted by the research team were then approached by the investigators and were again explained the objectives of the study. Afterwards, the investigators asked for their consent to participate. Key community informants were selected by workers of the PCC or by the project’s investigator of the PCC, who contacted them and forwarded the personal data of those who voluntarily and without coercion accepted to the interviewers. The project’s investigator of each PCC contacted with PHC workers to book them for group interviews. The decision of PHC workers to participate in the discussion groups and/or to recruit health-care users and key community informants was voluntary.

### Data collection and generation techniques

Conversational techniques were used: 11 discussion groups and 2 triangular groups (a meeting of 3 people to discuss a topic or issue with the aim of capturing the range and intensity of their views) [29] with health-care users; 30 semi-structured individual interviews with key community informants (15 health workers and 15 non health workers); and 14 discussion groups with PHC workers. Table 2 shows the characteristics of the 276 participants. The preliminary analysis of the information started simultaneously with the interviews and data relevance and richness was obtained.

**Table 2:** Description of participants according to region

Discussion groups with health-care users					
Region	Technique	Participants	Age	Gender	Educational level
Aragon	2 DG	20	9 between 45 and 59 years of age 11 from 60 to 75 years	10 women 10 men	13 primary education 7 secondary education
Balearic Islands	2 DG	13	6 between 45 and 59 years of age 7 between 60 and 75 years of age	6 women 7 men	9 primary education 3 secondary education 1 university education
Basque Country	2 DG	23	8 between 45 and 59 years of age 15 between 60 and 75 years of age	12 women 11 men	10 primary education 13 secondary education or more
Castilla-Leon	2 DG	16	3 between 45 and 59 years of age 13 between 60 and 75 years of age	10 women 6 men	12 primary education 3 secondary education 1 university education
Castilla-La Mancha	1 DG 1 TG	11	6 between 45 and 59 years of age 5 between 60 and 75 years of age	8 women 3 men	4 primary education 6 secondary education 1 university education
Catalonia	2DG 1TG	18	4 under 40 years of age ^a^ 6 between 45 and 59 years of age 8 between 60 and 75 years of age	9 women 9 men	9 primary education 3 secondary education 6 university education
Interviews to key community informants					
Region	Technique	Participants	Age	Gender	Occupation
Andalusia	3 SI	3 1 health worker 2 non health workers	2 between 50 and 59 years of age 1 between 60 and 69 years of age	1 woman 2 men	Representative of residents’ association Physician Educator
Aragon	5 SI	5 2 health workers 3 non health workers	1 between 30 and 39 years of age 2 between 40 and 49 years of age 2 between 50 and 59 years of age	1 woman 4 men	Paediatric nurse Specialist in internal medicine Responsible for social services Residents’ association president Secondary school teacher
Balearic Islands	4 SI	4 1 health worker 3 non health workers	1 between 40 and 49 years of age 2 between 50 and 59 years of age 1 between 70 and 75 years of age	1 woman 3 men	Social services coordinator Association for children, youth and family Pharmacist Representative of association for the elderly
Basque Country	5 SI	5 4 health workers 1 non health worker	1 between 30 and 39 years of age 1 between 40 and 49 years of age 3 between 50 and 59 years of age	2 women 3 men	Pharmacist Physiotherapist Primary care manager Physician Social worker
Castilla-Leon	4 SI	4 3 health workers 1 non health worker	1 between 30 and 39 years of age 3 between 50 and 59 years of age	3 women 1 man	Medical coordinator Pharmacist Council’s health technician Social worker
Castilla-La Mancha	5 SI	5 2 health workers 3 non health workers	2 under 40 years of age 1 between 40 and 49 years of age 1 between 50 and 59 years of age 1 between 60 and 69 years of age	3 women 2 men	Representative of the university for the elderly Medical coordinator Pharmacist Sports promoter Nursing coordinator
Catalonia	4 SI	4 2 health workers 2 non health workers	2 between 30 and 39 years of age 1 between 40 and 49 years of age 1 between 50 and 59 years of age	3 women 1 man	Physician Community pharmacist Council sports coordinator Careers service coordinator in community centre
Discussion groups with primary health care workers					
Region	Technique	Participants	Age	Gender	Occupation
Andalusia	2 DG	20	1 under 30 years of age 6 between 30 and 49 years of age 13 between 50 and 65 years of age	13 women 7 men	3 Administrative staff 4 Nurses 11 Physicians 2 Social workers
Aragon	2 DG	22	4 under 30 years of age 5 between 30 and 49 years of age 13 between 50 and 65 years of age	18 women 4 men	2 Administrative staff 8 Nurses 10 Physicians 2 Social workers
Balearic Islands	2 DG	20	7 between 30 and 49 years of age 13 between 50 and 65 years of age	14 women 6 men	3 Administrative staff 6 Nurses 9 Physicians 2 Social workers
Basque Country	2 DG	21	3 under 30 years of age 3 between 30 and 49 years of age 15 between 50 and 65 years of age	15 women 6 men	2 Administrative staff 8 Nurses 11 Physicians
Castilla-Leon	2 DG	18	1 between 30 and 49 years of age 17 between 50 and 65 years of age	12 women 6 men	4 Administrative staff 6 Nurses 8 Physicians
Castilla-La Mancha	2 DG	19	2 between 30 and 49 years of age 12 between 50 and 65 years of age 5 unknown data	14 women 5 men	2 Administrative staff 6 Nurses 1 Physiotherapist 8 Physicians 2 Social workers
Catalonia	2 DG	25	1 under 30 years of age 17 between 30 and 49 years of age 7 between 50 and 65 years of age	22 women 3 men	4 Administrative staff 10 Nurses 1 Nursing student 8 Physicians 2 Social workers

Technique: Discussion groups (DG); Semi-structured interview (SI); Triangular group (TG)

No discussion groups with health-care users took place in Andalusia

^a^ Women of the triangular group from the Maghreb

Interviews were based on a topic guide with some adaptations according to the type of informant (Appendix A). The topic guide was based on a literature review of HA and the salutogenic approach and on the experience of the research team on HP. The guide focused on three areas: 1) exploration of health only in health-care users and key community informants, and HP concepts in all participants; 2) actual HP practice in PHC; and 3) proposals to approach HP in PHC. The first area is reported in this paper and the third has already been published [26]. The topic guide was pilot tested with each group of participants. Semi-structured individual interviews took place in a setting accessible for the informants and had a duration of 45–60 min. The discussion groups took place in the PCC with one moderator and one observer, and lasted between 90 and 120 min. After obtaining informed consent from the participants, the interviews were recorded in audio or video with the exception of the triangular group of women from the Maghreb. This group of women, resident in Catalonia, did not consent to the recordings and thus only notes were taken. The field work was carried out by interviewers, who followed the manual that standardized the procedures. At the end of each interview a summary with the key ideas was written down. All interviews were conducted in Spanish or Catalan. Data collection took place between November 2013 and May 2014.

### Analysis of the information

All recordings were transcribed verbatim; the data that identified informants were anonymized. The transcriptions were carried out by experts and reviewed by the interviewers. In relation to the concepts of health and HP, a thematic interpretive content analysis was carried out [30, 31] with the support of Atlas.ti software. Preanalytical intuitions were formulated after successive readings of the transcriptions and the observation notes. Four investigators (AB, NCA, MPV and EPR) discussed and created an initial analytical plan and performed the text codification. Subsequently, one investigator of each region independently analysed the data from the key informant participants (AAM, HPR, MMA, PMP, SM and SM). Afterwards, four investigators (AB, NCA, MPV and EPR) created categories classifying the codes according to the criterion of analogy following the pre-established analytical criteria in the objectives of the study and new elements from the comments’ codes. Finally, the meanings from each type of informant were interpreted separately, and subsequently a joint comparative analysis was carried out. The whole analysis was an iterative process and the four investigators (AB, NCA, MPV and EPR) discussed discrepancies and examined them until reaching consensus.

In relation to HA and deficits within the neighbourhood, the items identified by the various types of informants were listed and later classified according to the asset mapping proposal of Botello et al [32], an adaptation of the Improvement and Development Agency [33] that classifies resources in 6 large groups: resources of individuals; of formally and non-formally established associations; physical resources in the area; financial; cultural; and finally, the resources of the organizations. Later, a comparative analysis was carried out to detect differences in the assets and deficits identified by the 3 types of informants.

### Rigour and quality criteria

We adhered to the following rigour criteria suggested by various authors [34]: description of context, of participants (selection strategies of participants) and of the research process (information generating procedures; procedures for the analysis and saturation of information); adequacy between research questions and methodology used; data triangulation (sources and techniques); reflexivity of the interdisciplinary research team throughout the whole research process; prior assumptions of researchers and analysis of possible influences on their investigation; illustration of results with relevant quotations that support the interpretation; and use of the field notebook to enhance reflexivity and validity.

## Results

The findings of the analysis are divided into 5 main categories: concept of health according to health-care users and key community informants; concept of HP according to all participants; HP in the daily life of the health-care users; HA of the neighbourhood; and deficits of the neighbourhood. Quotations from discussions with the 3 different types of participants are included to illustrate the process of interpretation based on these data. These quotations were translated by a professional scientific translator and later reviewed by the research team to verify that the meaning of the original discourse was maintained.

### Concept of health according to health-care users and key community informants

All informants’ profiles consider that the concept of health is complex, broad and multifactorial. They define health according to their own experiences of health and disease and the social determinants that shape these experiences.

*What can I say, it is a difficult question, health is very difficult to define… (Woman, key informant, Castilla-León)*

For health-care users and key community informants, health is the first, most fundamental aspect of life. Without health, everything else appears irrelevant. They explain that health is a dynamic concept, that it constitutes more of a concern with age and that it is taken for granted until a health condition arises.

*Health is very important. It’s the first thing we need to take care of, for us and for our children. Without health we cannot live (Woman, health-care user, Catalonia)*

*So what can I say, it is one of the main axes of…of the life dynamics of a person, isn’t that so? It’s one of the main things… (Man, 43 years, key community informant, Basque Country)*

*Health is more appreciated when you don’t have it that when you feel well, because when you are healthy you don’t pay any attention to it. (Health-care user, Aragon)*

The concept of health emerged from the analysis of the responses represents a continuum that starts from health described in negative terms (absence of disease, absence of pain and not requiring medication) and reaches a more complex definition that includes several dimensions beyond the bio-psycho-social concept. These dimensions, namely physical, psychological-emotional, social, occupational, intellectual, spiritual and environmental, are interrelated to achieve a state of equilibrium. The three last dimensions (intellectual, spiritual and environmental) appear only occasionally in the participants’ discourses. Every person can be located at some point of this continuum in a position more oriented toward one of the dimensions and its interrelations. Within this continuum they talk about wellbeing, happiness, absence of worries, balance, an active life, autonomy, quality of life, one’s own approach to life, living each day to the full and participation in activities. A key community informant even mentions fate. While most participants highlight the physical and psychological dimensions, they also refer to the social dimension of health, which is particularly emphasized by the key community informants.

*I for me is not feeling any pain… you cannot do what you previously did because of the pain you feel. It affects me mentally because it generates emotional stress (Health-care user, Castilla-La Mancha)*

*In my opinion, physical health is as important as psychological health, sometimes one causes the other and vice-versa (Man, 54 years, health-care user, Aragon)*

*Health for me? Well, health is as…the wellbeing of a person from an integral perspective. I mean, not only health at a physical level, but also emotional health, psychological health, social health, isn’t that so? (Woman, key informant, Catalonia)*

Many answers refer to the social determinants that influence how people live and how they feel. These determinants focus on the family, the immediate environment and the financial and occupational situation. Family is a factor that impacts on health and it is also a great motivator to take care of one’s own health.

*For me it’s the situation of my family. The people around me, I make their problems my own, and then I enter a loop of anxiety and this anxiety, I admit, is going to the biscuit tin, to the fridge, to get nuts. (Woman, health-care user, Castilla-León)*

*…. a health state, completely free of disease is impossible… Many factors have an impact, heredity, age, socioeconomic status, and a primary care that is important, and I believe that too often it doesn’t exist. (Woman, key informant, Castilla León)*

### Concept of health promotion according to health-care users, key community informants and PHC workers

While HP is unquestionably relevant and indispensable for all participants’ profiles, their definitions of HP are complex and vague. Participants express different meanings that encompass the implementation of preventive activities and follow up of existing health problems, health education, starting and sustaining healthy behaviours and empowerment and self-management (integral concept that relates the individual with her environment and lifestyle). All profiles agree that HP behaviours cannot solely originate from the health services, but must be built through a process of community participation with the contributions of the educational, politics and social sectors.

*What we understand by promotion? that it’s different from prevention, which is what we are mixing up… (Woman, 47 years, physician, Balearic Islands)*

*Well, a health promotion behaviour is a behaviour that improves lifestyles, that facilitates lifestyles that are good for health, that achieve that people take more responsibility over their own health and that are in some way related with achieving a better health level for the population. (Key informant, Castilla León)*

*It is having an active life, taking care of oneself, eating reasonably well, avoiding excess… (Man, 69 years, health-care user, Balearic Islands)*

*Because promoting health promoting behaviour from a health centre, well it seems something we should wish for. But when you involve various agents, the pharmacy, associations, the teachers or parents at school, the social workers, eventually they contribute to build a network that goes beyond a promotion of … I mean, I believe that this should work as a group of people that push the others. It has to be participative (Man, 46 years, pharmacist, key informant, Basque Country)*

PHC workers highlight health education activities within HP: explaining healthy habits (specifically eating, exercise and self-care) and making the public responsible for their own health. The key community informants highlight the social, emotional and self-awareness aspects of HP. Health-care users identify HP with getting information and advice from health professionals, following the professionals’ recommendations, carrying out preventive activities and taking care of interpersonal relationships.

*Behaviours that facilitate the health status of the population, of my patients. Behaviours that help the patient understand the most important things to take into … account and that need to be implemented for a healthy life. From the point of view, not only physical, but also emotional and spiritual (Woman, 53 years, health care key informant, Basque Country)*

*Behaviours that facilitate keeping or improving health, they are not exactly related with lifestyle. I believe that this health promotion behaviour is much more than that. It is a way of understanding life and finding your place in it. Then it would be… more than instructions to the patient it would be creating a certain social climate where I think that community intervention is much more important than an intervention case by case… (Woman, 57 years, key informant, Andalusia)*

### Health promotion in daily life according to health-care users

Most health-care users explain that they have implemented some **change to promote their health**. Physical activity, healthy eating and quitting smoking are the most commonly reported changes. They also explain that it is important on occasion to treat themselves. Contentment is also considered to be part of health. Other activities related to HP are reading, sewing, learning information technology skills and most of all socialising, either through planned activities, talking to people in the street or meeting friends.

*I encourage everybody to join a walking group: contact with nature, walking, socialising, and within the group there are always subgroups of people with whom you immediately connect and where you feel extremely comfortable … that’s what I need. (Health-care user, Castilla-León)*

*What I do? In the morning, I go to the soup kitchen of Caritas [NGO], and in the evening I go to the shelter to help with dinner…. I attend English lessons twice a week; I go dancing with my wife; every single day I walk 6 km … (Health-care user, Castilla-La Mancha)*

Having a disease or the fear of contracting a disease, prevention and taking care of oneself in order to be healthy are the **main motives to implement changes in lifestyle**. Family, trust in PHC workers, psychological assistance, group activities, social network and friends are all facilitators of change. In addition, health-care users underscore the importance of being determined and realising that you feel better after the changes, because it encourages you to keep going.

*that I was well, simply I felt pathetic 1 day, I left home in my pyjamas to get cigarettes, at two a.m. and I thought, this is bad, you cannot continue like this, and in terms of health I was very well, nobody ever told me quit smoking, but I thought—what am I doing, getting in the car to get cigarettes. Then I thought, I’ll quit, it’s like a click that happens to you, I don’t want to keep smoking, I don’t want to live with this for the rest of my life, and it was not easy, eh! Not easy, that’s why I joined the group and it was hard for me. (Woman, 51 years, health-care user, Balearic Islands)*

*I really quit smoking due to chest pains, I had chest pains and because of that I had arrhythmia, I also had apnoea, in short, everything was related to smoking and then, naturally, I got fear and of course in relation to that I had to stop, well, I’m telling you that during 1 year, I was quitting for the whole year, it was very hard but finally I succeeded. (Man, 59 years, health-care user, Balearic Islands)*

Health-care users and PHC workers readily admit that it is **difficult to implement and sustain changes**. Despite being concerned about health and admitting that they should implement changes, health-care users point at lack of determination or motivation as the main difficulty. This lack of determination is closely related with excuses that prevent them to put into effect healthy behaviours, for instance: weather conditions for physical activity, a culture of bad habits (social life implies drinking alcohol, eating heavy foods and eating too much), lack of time because of work, stress at work, life-stress events, family burden and boredom at home that makes you eat worse. Some add that they have little information on the benefits of changing and therefore find it difficult to follow advice.

*This very basic measures are sometimes very difficult. Things like…eating well, avoiding alcohol, all these things that we all know we have to do … they are very difficult to do. That’s the million-dollar question. Why is it so hard? (Woman, 52 years, physician, Basque Country)*

*Some behaviours we don’t stop because they are not only something personal, but part of the group. I say group because, well, it also depends a bit of how the social life of each of us is, isn’t it? Or how it’s connected with our environment. (Man, 61 years,* *health-care user, Basque Country).*

*You have to walk but then, I started at 9 a.m. and finished at 8 or 9 in the evening and naturally, I couldn’t start exercising being that exhausted. (Man, 52 years, health-care user, Aragon)*

Health-care users think that **information** is crucial to understand which HP activities they should undertake and how to put them into practice. Some say that information comes basically from PHC workers, but sometimes health-care users only get the information when they are already unwell and feel they should have been informed before. They also look for information on the internet, magazines, with friends and on tv, in particular when they have a specific problem and feel worried about it.

*I look for information on the internet, the consultation is very brief and they do not provide much information (Woman, 37 years, health-care user, Catalonia)*

*I believe that there is a lot of information… if you pay attention they warn us on TV …these campaigns of vegetables, fruit, the importance of the Mediterranean diet. We hear and watch about it every day … (Woman, 57 years, health-care user, Basque Country)*

### Health assets and deficits in the neighbourhood in relation to health promotion behaviour

Tables 3 and 4 show the assets and deficits of the neighbourhood in relation with HP behaviour. The objective is to compare agreements and discrepancies between the different profiles of participants. The results show that the three groups coincide in many aspects. However, key community informants and PHC workers are generally more in agreement, with the exception of the stronger identification of social aspects in the case of key community informants (such as participation in activities and volunteering) and of resources related to provision of healthcare in the case of PHC workers. In contrast, health-care users focus specially in practical resources for everyday life.

**Table 3:** Neighbourhood assets related to health promotion behaviours as identified by the three groups of participants

NEIGHBOURHOOD ASSETS			Key community informants	Primary Health Care workers	Health-care users
Resources of individuals			social networks multiculturality-diversity neighbourhood action participation/voluntary work immigrants word of mouth feeling of belonging young people with children attend regularly the HC	family and neighbourhood network adherence to programmes respect for the doctor own beliefs immigrants like a village	neighbourhood network good environment/antidepressant/entertainment proactive people active neighbourhood good neighbourhood willing to work young neighbourhood/young people new neighbourhood poor neighbourhood
Associations			associations neighbour’s association women’s association AMFS	associations neighbour’s association patient’s association	associations neighbour’s association
Physical space	Natural resources		river, sea, beach rural area ecologic orchards	river plain orchard	river, beach plain
	Infrastructures	Urbanism	park/green space walk/healthy walk gym equipment/cycle track	park/green space healthy walk	park/green space/gardens walk/healthy walk gym equipment
			public sports centre	leisure areas	leisure areas
			indoor swimming pool		
			health centre community centre schools/nursery school library cultural centre day-care centre	health centre schools library community centre for the elderly day-care centre care home detoxification centre church	health centre community centre schools library community centre for the elderly social centre
			close to town centre	close to town centre close to the hospital	close to town centre close to the hills
		Housing	well equipped	no elevator (exercise)	
	Transport and mobility		well communicated everything is close	well communicated	well communicated
	Environmental characteristics		town peace and quiet peaceful spots flat areas	no pollution peace and quiet	no pollution peace and quiet/not noisy clean area sun, air, climate
Cultural and sports activities			free internet arts and crafts IT activities for young people gym activities cooking	cinema dancing memory workshop	cinema dancing concerts theatre talks courses Pétanque aqua-gym
Organizations’ Resources			city council- activities library promotion of sport low-cost activities cultural centre community centre-activities health centre-activities food banks/soup kitchen social worker of the HC relationship between institutions	city council- activities library swimming pool gym	city council- activities university of the third age swimming pool gym cultural centre exhibitions hall health professionals
Socioeconomic aspects	Financial resources		market/solidarity markets	market	market
			shops		cafeteria
			private sports centre pharmacies not expensive	shops private sports centre	private sports centre
	Socio-economic factors		high socioeconomic level mid-high cultural level working-class neighbourhood thankful population	high socioeconomic level cultural level educational level	working-class neighbourhood poor neighbourhood

*AMFS*: association of mothers and fathers of students; *HC*: health centre; *IT*: Information Technology

**Table 4:** Deficits of the neighbourhood related to health promotion behaviours as identified by the three groups of participants

NEIGHBOURHOOD DEFICITS			Key community informants	Primary Health Care workers	Health-care users
Resources of individuals			elderly individualism immigration cultural differences marginal population groups	elderly take care of grandchildren immigration own beliefs bad habits population prone to litigation floating population	elderly individualism it’s a village
Associations			Poor neighbourhood network; Poor social cohesion		
Physical space	Natural resources		cold climate		dampness lack of sunshine
	Infrastructures	urbanism	very high buildings lack of green space	industrial estate lack of parks structural barriers empty houses empty building sites	very high buildings old neighbourhood
		housing	dampness poor quality very old	poorly adapted in bad condition no elevator	
	Transport and mobility		too many cars-gases	too many cars	too many cars/traffic/traffic jams
	Environmental characteristics		massification dirt neglected area foul smells factory car pollution	dirt animals car pollution	massification noise (train, rubbish, dogs, children, neighbours,etc) pollution (air, water..), cars dirt/dog’s excrement
Cultural activities			lack of activities for children no options to occupy free time activities that require payment		
Organizations’ resources			HC/professionals lack coordination HC-org bureaucracy lack of awareness resources	HC/professionals lack coordination HC-org worse health resources lack of awareness resources	few places for activities corruption town police few police personnel
Socioeconomic aspects	Financial resources		↑ bars-normalisation of alcohol junk food	↑ bars-normalisation of alcohol	↑ bars-normalisation of alcohol it’s expensive
	Socioeconomic factors		unemployment socioeconomic problems financial level and crisis low cultural level drugs long working hours ICT-only young people	unemployment socioeconomic problems financial level low cultural level drugs social exclusion isolation/limitations	unemployment

*HC*: *Health Care*; *HC-org: health centre with other organisms*;* ICT*: information and communication technology

↑ bars-normalisation of alcohol: lots of bars

Health-care users report as assets of the neighbourhood people that is hard working and willing to take part in activities. Those that live in a working class or poor neighbourhood feel proud about it, which shows a feeling of belonging. In addition, they think that good neighbourly relations are important. Key community informants and PHC workers highlight the cultural and socioeconomic level, considered an asset when high and a deficit when low. Both report also socioeconomic problems and drug abuse as negative elements. Another element identified as an asset and deficit by key community informants and PHC workers is immigration. Some consider that multiculturality and diversity benefit the neighbourhood. In contrast, others consider that they make their work more difficult and associate them with a marginal, floating population that decreases the stability of the neighbourhood.

With regard to the health centre, the key community informants underscore that people attend it regularly. For the PHC workers, the respect of the health-care users is essential. In relation to physical space, key community informants and PHC workers identify orchards as an asset. In contrast, health-care users emphasize the noise and pollution of air and water. With reference to infrastructures, participants concur in most assets (green spaces, schools, library, community centre), but PHC workers mention more facilities related to health services (care home, detoxification centre, pharmacy and proximity to hospital), as well as the church. In addition, PHC workers report more negative aspects such as environmental barriers, unoccupied housing and empty building sites. The market is amongst the most valued assets by the three profiles of informants; health-care users are proud of the market and point at the cafeterias as hubs of socialization.

In relation to the resources of organizations, the main differences are in the deficits. Health-care users underscore the corruption and that too few places are available for the activities on offer. Key community informants and PHC workers identify as deficits the poor coordination between the health centre and the different organizations of the neighbourhood, the consequences of the health cuts and the lack of awareness of available community resources. In addition, key community informants think that there is too much bureaucracy.

Interestingly, whereas health-care users do not mention any aspect of housing, key community informants and PHC workers refer to the poor condition of some as a deficit. PHC workers point out at the lack of elevator as an asset because it makes you exercise, but also as a deficit because of the isolation and difficulties the absence of elevator implies.

## Discussion

Health-care users and key community informants agree that health is a complex, broad, multifactorial concept that encompasses several interrelated dimensions (physical, psychological-emotional, social, occupational, intellectual, spiritual and environmental). The three participants’ profiles consider HP indispensable despite defining it as complex and vague. In fact, most health-care users admit to having implemented some change to promote their health. The most powerful motivators to change lifestyles are having a disease, fear of becoming ill and taking care of oneself to maintain health. Health-care users believe that the main difficulties are associated with the physical, social, working and family environment, as well as lack of determination and motivation. They also highlight the need for more information. With regard to HA and deficits, the three groups of participants coincide in many aspects. However, the highest agreement is found between key community informants and PHC workers, although key community informants emphasize social aspects whereas PHC workers underscore the resources related to health care. In contrast, health-care users focus on the practical resources for everyday life.

The definition of health of most health-care users and key community informants is precise, associated with multiple interrelated dimensions that include a collective vision, and consistent with the current approaches to health [23, 35], similarly to the positive concept of health of professionals and health-care users in the study of Hunter et al [25]: “Health is more than a physical and psychological wellbeing, there are many attributes associated like happiness, life satisfaction, cognitive capacities, spiritual, social, occupational wellness and environmental”. Even though participants refer to all these dimensions, the emphasis is lower on the spiritual, environmental and intellectual aspects. Nevertheless, they associate wellbeing, balance, autonomy and happiness with the concept of health in agreement with the definition of Jordi Gol [5]. Health-care users and key community informants provide a large number of factors with an impact on health that coincide with their social determinants [36], confirming the integral and social view of health of the population. Indeed, people are aware of the collective dimension of health and that the responsibility for this collective dimension lies beyond the individual [37]. We agree with Johansson et al [2] that there is a discrepancy between the holistic concept of health communicated by the participants and the actual practice of the health services, which still views health as the opposite of disease. The health system has prioritized specialisation and division of tasks. As a result, current health practice is fragmented, disease-centered and focused on problem solving [37]. We concur with Barbara Starfield’s view that to achieve more effective, efficient, safer and equitable primary care services, the emphasis should shift from treating diseases to caring for individuals and populations [38]. PHC plays a key part in addressing the social determinants of health, mainly through its role in the community, and contributing, in collaboration with other sectors, to the reduction of social inequalities in health [39]. Action for integrating social determinants of health into PHC practice should therefore be prioritised [40]. In addition, global conferences on health promotion emphasize the inclusion of the needs, values and views of the population in all health policies.

It is harder for the three participants’ profiles to define the concept of HP, which they associate with very diverse actions, activities and strategies. In agreement with previous studies, we observed that despite a positive attitude and awareness of its importance, the definition of HP conveys a more traditional, individual approach and is equated to prevention and health education [24, 41]. This description is not consistent with the tenets of the salutogenic interpretation of the Ottawa Charter, which is based on the values of equity, participation and empowerment [42]. The difficulty in implementing HP recommendations based on the salutogenic paradigm and HA might contribute to the participants’ more traditional and individual approach to HP. Nonetheless, the three groups of informants explain that HP is not exclusively the responsibility of the health services, which shows their understanding of the impact of policies on health and of intersectoriality in HP [43]. These results show that both the public and the professionals agree in the need for this paradigm shift, supporting the implementation and sustainability of the new approach.

PHC is the ideal setting to advance HP. However, patients have reported low rates of lifestyle advice in PHC in previous studies [21, 22]. We believe that a more effective HP should be planned according to the needs of the target population and aimed specifically at community health. In addition, the implementation must take into account the barriers and facilitators of behavioural change and HP reported by the health-care users of this and previous studies. A review of the literature concludes that health-care users and PHC professionals [9, 10] have identified intrapersonal, interpersonal, institutional, environmental and social factors for the successful implementation of HP. Equity constitutes a fundamental element when designing and implementing the interventions to avoid just benefiting those in less need and consequently increasing social inequities [44].

To obtain an exhaustive asset mapping [12, 32, 45] of the community resources, the views of the various collectives of that specific environment should be taken into consideration. Asset models emphasise the positive capabilities of people and communities and encourage tackling the issues of inequity in health with the active participation of communities. Asset identification must be linked to the design of HP community activities and should be the source of social prescription by PHC professionals. It is important to underline that PHC workers tend to be more aware of the resources related to health services. There is thus a need for a broader participation in asset mapping with the health-care users at the centre. On the other hand, asset mapping requires trained professionals and resources. Ultimately, the current concept of HP based on salutogenesis, empowerment of the population and community advance involves working with HA within and outside the consultation room [5]. Supporting this vision in the culture of HP and community health is in essence more important than the actual asset mapping [46].

The process of identification of assets and deficits with group interviews is a data collection technique that does not necessarily equate with qualitative methodology. Beyond the identification and listing of resources, qualitative research implies a more interpretive analysis that looks for agreements and discrepancies amongst the different profiles of participants in accordance with the objectives of this research.

### Strengths and limitations of the study

One of the strengths of this study is the broad selection of discourses on the meaning of health, HP, assets and deficits obtained through the participation of three types of informants in 7 diverse Spanish regions. A deep understanding of these meanings through the participation of different stakeholders is essential for the design of successful, acceptable, equitable, feasible and sustainable HP strategies that are adapted to their context. Indeed, this study has been conducted as part of the EIRA Project and the results will be incorporated in the design and implementation of a complex, multi-risk intervention to develop health-promoting behaviours. Moreover, one of the ten principles for policy action towards advancing in health equity is to make concerted efforts to facilitate people’s participation in decisions that affect their health [47]. On the other hand, the consultation of the population constitutes a first step in the health-care users’ involvement in HP research; it also represents a novel approach for research in our setting. In addition, alongside health-care users our study includes members of the public without any particular health condition. Most published articles have been conducted in a hospital setting or in populations with a specific disease. The salutogenic approach and the asset model is an emerging research topic and a challenge for the present and the future in PHC and in public health.

Even though the design included a theoretical sampling, participant workers in PHC centres volunteered to take part in the EIRA project, which suggests a particular interest in HP. Consequently, caution should be applied with regard to the attitude of this collective toward HP, which might not be transferable to other more sceptical professionals.

Scheduled meetings and a researcher’s manual guaranteed uniformity of techniques implemented by different interviewers in each region. Sample sufficiency was attained with the richness and complementarity of the information generated by the different techniques with the three types of participants from 7 regions. The rigour procedures used (triangulation of techniques and analysis, description of context, working with different actors, theoretical sampling, reflexivity and interdisciplinary research team) ensured the validity of the findings. Although caution is needed before transferring these results to other settings, the similarity with other studies suggests its applicability. On the other hand, the participation of immigrants in the exploration of health and HP was on this occasion limited to a triangular group of women from the Maghreb. This group’s opinions highlighted the need to include the views of immigrants and of the most disadvantaged members of society. We also tried to capture their discourse and opinions through key community informants.

Although the analysis of perspectives by gender, age and professional profile in the case of PHC workers was not an objective of the current investigation, we consider that further analyses taking into account this stratification would provide valuable information. Finally, this study analyses only the first of three parts of the interview. We noticed that despite the richness of the discourses, the emerging concepts of the debate could have been further explored if the interviews had exclusively focused on this first section.

## Conclusions

This study explores the concept of health and HP and associated activities and compares HA and deficits as identified by health-care users, key community informants and PHC workers in 7 Spanish regions. Although participants express a holistic and positive concept of health, they manifest a more traditional and individual approach to HP, which they find harder to define. It is therefore crucial to shift the practice of health services toward HP, wellbeing and community participation to depart from the approach focused on the individual and the disease and to substantially increase the participation of every citizen. Effective implementation strategies to translate theory into practice become thus essential to advance the cause of HP based on the paradigm of salutogenesis and HA. Finally, further research should address HP processes founded on the assets model and salutogenesis.

## Appendix A

**Table 5:** Topic guide for the data generation techniques according to type of informant

Health-care users (object of the intervention)	
Exploration of meanings	For you, what does “health” mean? For you, what does “behaviours that promote health” mean?
Neighbourhood assets Assets for health promotion	Positive aspects of the neighbourhood with regard to health promotion activities (structural, cultural and human resources) Negative aspects of the neighbourhood with regard to health promotion behaviours
Source of information for participants on health promotion	Have you received information on this topic? Who did provide you with information?
	Have you looked for information on this topic? Where did you look for information?
Concerns of informants in relation to their health. Activities, resources and difficulties for health promotion	How important are health promotion behaviours for you?
	What worries you about your health? (brief intervention of all participants)
	What do you do to keep and promote your health? (individual, interpersonal and community level)
	Which resources do you have to put into effect healthy behaviours? (Identify facilitators of healthy behaviours)
	What difficulties do you face to put into effect healthy behaviours? (Identify the elements that interfere with healthy behaviours)
	What else could you do to improve your health?
Key community informants (with in-depth knowledge of the context and the population object of the intervention)	
Exploration of meanings	For you, what does “health” mean? For you, what does “behaviours that promote health” mean? For you, how important are health promotion activities?
Neighbourhood assets Assets for health promotion	Positive aspects of the neighbourhood (structural, cultural and human resources) Positive aspects of the neighbourhood in relation to health promotion behaviours. Negative aspects of the neighbourhood Negative aspects of the neighbourhood in relation to health promotion behaviours
Primary health care professionals	
Exploration of meanings	What does “health promotion behaviours” mean to you? If you had to explain the meaning of “health promotion behaviours”, what would you say?
	How important is for you to encourage health promotion activities?
Neighbourhood assets Assets for health promotion	Which positive aspects does the neighbourhood have? (structural, cultural and human resources) Which negative aspects does the neighbourhood have? Which positive aspects does the neighbourhood have in relation to health promotion behaviours? Which negative aspects does the neighbourhood have in relation to health promotion behaviours?

## Abbreviations

HA: Health assets
HP: Health promotion
PCC: Primary Care Centre
PHC: Primary Health Care
